# Defeated by the nines: nine extracellular strategies to avoid microbe-associated molecular patterns recognition in plants

**DOI:** 10.1093/plcell/koab109

**Published:** 2021-04-19

**Authors:** Pierre Buscaill, Renier A. L. van der Hoorn

**Affiliations:** The Plant Chemetics Laboratory, Department of Plant Sciences, University of Oxford, OX1 3RB Oxford, UK

## Abstract

Recognition of microbe-associated molecular patterns (MAMPs) by cell-surface receptors is pivotal in host-microbe interactions. Both pathogens and symbionts establish plant-microbe interactions using fascinating intricate extracellular strategies to avoid recognition. Here we distinguish nine different extracellular strategies to avoid recognition by the host, acting at three different levels. To avoid the accumulation of MAMP precursors (Level 1), microbes take advantage of polymorphisms in both MAMP proteins and glycans, or downregulate MAMP production. To reduce hydrolytic MAMP release (Level 2), microbes shield MAMP precursors with proteins or glycans and inhibit or degrade host-derived hydrolases. And to prevent MAMP perception directly (Level 3), microbes degrade or sequester MAMPs before they are perceived. We discuss examples of these nine strategies and envisage three additional extracellular strategies to avoid MAMP perception in plants.

## Teaser

This review discusses and illustrates nine extracellular strategies that plant pathogens and symbionts use to avoid the recognition by cell surface receptors.

## Introduction

Plants are a rich source of nutrients for many organisms (Cardinale et al., 2011). Their root and aerial systems are exposed to a wide range of microbes including bacteria, fungi, and oomycetes. Some of these microbes are plant pathogens that cause detrimental effects on plant fitness (Dangl and Jones, 2001; Dodds and Rathjen, 2010), while symbiotic microbes can promote plant growth, such as rhizobacteria and arbuscular mycorrhizal fungi (Pérez-de-Luque et al., 2017).

When microbes enter the extracellular space within plant tissues (the apoplast), they interact with host cells that carry surface receptors that recognize conserved molecules, conventionally called microbe-associated molecular patterns (MAMPs, Jones and Dangl, 2006). MAMPs are fragments of proteins or glycans that are essential for the biology of microbes and are absent in the host plant. Examples are fragments of flagellin and peptidoglycans (PGN) from bacteria and fragments of chitin and β-glucans from fungi and oomycetes. These MAMPs are recognized on the host cell surface by pattern recognition receptors (PRRs). PRRs are transmembrane receptor-like kinases (RLKs) or receptor-like proteins (RLPs) that often carry extracellular leucine-rich repeat (LRR) or lysine motif (LysM) domains to confer MAMP recognition (Couto and Zipfel 2016; Tang et al., 2017; Boutrot and Zipfel, 2017; Schellenberger et al., 2019). PRR activation triggers a series of immune responses (Bigeard et al., 2015), resulting in pattern-triggered immunity (PTI) and preventing colonization by nonadapted microbes (Bigeard et al., 2015; Andersen et al., 2018). Importantly, many MAMPs require hydrolytic release from their precursors before they can be perceived by PRRs. For instance, hydrolytic release from precursors is required for the recognition of flagellin and elongation factor Tu (EF-Tu), and for the recognition of chitin and β-glucan.

**Figure 1:**
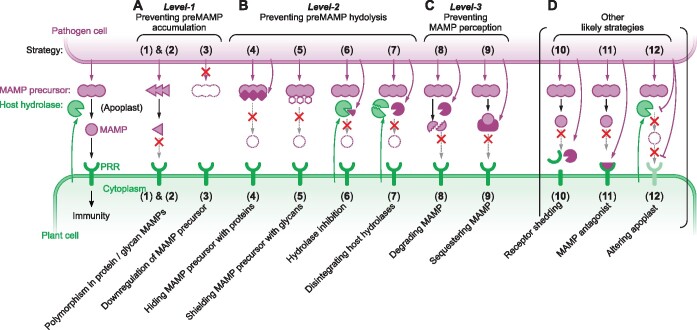
Nine known and three possible strategies to evade extracellular recognition in plants. Illustration of microbial strategies that evade extracellular recognition (A) by preventing the accumulation of MAMP precursors (Level 1, Strategies 1–3); (B) by preventing the hydrolysis of MAMP precursors (Level 2, Strategies 4–7); and (C) by preventing MAMP perception (Level 3, Strategies 8 and 9). (D) Other possible but as yet unreported strategies. MAMP precursors, MAMPs, and other microbial molecules are colored in purple, and host hydrolases and receptors in green. Secretion by microbe and plant are represented by arrows in purple and green, respectively.

To colonize plants, adapted pathogens and symbionts have adopted strategies to avoid recognition by the immune system. Many effector proteins that act inside the host cell interfere in signaling downstream of PRRs (Toruño et al., 2016). In this review, however, we describe nine extracellular strategies that plant pathogens and symbionts use to avoid recognition by PRRs (Table 1). These strategies occur at three levels: MAMP production (Figure 1A), MAMP release (Figure 1B), and MAMP perception (Figure 1C).

**Table 1:** Overview of strategies employed by microbes to evade MAMP recognition in plants

Microbe		MAMP or MAMP precursor	Microbial protein	Plant host	Host target (PRR)	References	
Level 1: Preventing MAMP production							
Strategy 1: Polymorphisms in protein MAMPs							
Bacteria	*Agrobacterium tumefaciens*	flg22	N/A	Arabidopsis	(FLS2)	Felix et al. (1999)	
*Ralstonia solanacearum*		Arabidopsis	Pfund et al. (2004); Wei et al. (2018)				
*Xanthomonas campestris* pv. *campestris*		Arabidopsis	Sun et al. (2006)				
*Xanthomonas oryzae* pv. and pv. *oryzicola*		rice	Wang et al. (2015)				
*Xanthomonas arboricola* pv. *juglandis*		*Juglans regia*	Cesbron et al. (2015)				
*Sinorhizobium meliloti*		tomato, rice	Felix et al. (1999)				
*Burkholderia phytofirmans*		grapevine	Trdá et al. (2014)				
*Pseudomonas syringae* pv. *tomato* Col338		flgII-28		tomato	(FLS3)		Cai et al. (2011)
*Xanthomonas campestris* pv. *campestris* and *Pseudomonas syringae* pv. *tomato*		elf18	N/A		Arabidopsis	(EFR)	Lacombe et al. (2010)
*Xanthomonas oryzae* pv. *oryzae*		RaxX	N/A		rice	(Xa21)	Pruitt et al. (2015)
Strategy 2: Polymorphisms in glycan MAMPs							
Fungi	*Verticillium dahliae*	Chitin	*Vd*PDA1	cotton	(CERK1)	Gao et al. (2019)	
*Puccinia striiformis* f. sp. *tritici*		Pst13661	wheat		Xu et al. (2020)		
*Puccinia graminis* f. sp. *tritici*		N/A	wheat		El Gueddari et al. (2002)		
*Uromyces fabae*		N/A	broad bean				
*Colletotrichum graminicola*		N/A	maize				
*Pestalotiopsis* sp.		*Pes*CDA	rice		Cord-Landwehr et al. (2016)		
Strategy 3: Downregulating MAMP production							
Bacteria	*Pseudomonas*	Flagellin	cdG	N/A	(FLS2)	Hickman and Harwood (2008)	
*Pseudomonas syringae*		*N. benthamiana* and Arabidopsis	Pfeilmeier et al. (2016)				
*Pseudomonas aeruginosa*							
*Pseudomonas fluorescens*							
*Pseudomonas syringae* pv. *tomato*	AlgU	*N. benthamiana*		Bao et al. (2020)			
*Pseudomonas syringae* pv. *syringae* B728a		*fliC*		bean	Yu et al. (2013)		
Fungi	*Colletotrichum graminicola*	β-glucan	*KRE5* and *KRE6*	maize	N/A	Oliveira‐Garcia and Deising (2016)	
Strategy 4: Hiding MAMP precursors with proteins							
Fungi	*Cladosporium fulvum*	Chitin	Avr4	tomato	(CERK1)	Van den Burg et al. (2006)	
*Verticillium nonalfalfae*		VnaChtBP	hop (*Humulus lupulus* L.)		Volk et al. (2019)		
*Verticillium dahliae* strain VdLs17		Vd2LysM	tomato		Kombrink et al. (2017)		
*Colletotrichum higginsianum*		ChELP1 and ChELP2	monocot and dicot crops		Takahara et al. (2016)		
*Parastagonospora nodorum*		SnTox1	wheat		Liu et al. (2016)		
*Clonostachys rosea*		Cell wall	LysM1 and LysM2		wheat	Dubey et al. (2020)	
*Serendipita indica*		β-glucan	FGB1		Arabidopsis, barley, and *N. benthamiana*	Wawra et al. (2016)	
Strategy 5: Shielding MAMP precursors with glycans							
Bacteria	*Pseudomonas syringae* pv. *tabaci* 6605	Flagellin	flagellin glycan	tobacco	N/A	Taguchi et al. (2006)	
*Pseudomonas syringae* pv. *glycinea* race 4		soybean	Takeuchi et al. (2003)				
*Xanthomonas campestris* pv. *campestris* XcA		cabbage	Ichinose et al. (2013)				
*Acidovorax avenae* K1 strain		rice	Hirai et al. (2011)				
*Pseudomonas syringae* pv. *syringae* B728a		*N. benthamiana*	Buscaill et al. (2019)				
*Xylella fastidiosa*	LPS	LPS glycan	grapevine	N/A	Rapicavoli et al. (2018)		
*Pseudomonas syringae* pv. *syringae* B728a	bean		Helmann et al. (2019)				
Fungi	*Magnaporthe oryzae*	Chitin	α-1,3-glucan	rice	N/A	Fujikawa et al. (2009)	
*Cochlioborus miyabeanus*		Fujikawa et al. (2012)					
*Rhizoctonia solani*							
Strategy 6: Blocking MAMP release by inhibiting the activity of host hydrolases							
Oomycete	*Phytophthora sojae*	β-glucan	GIP1	soybean	EGaseA	Rose et al. (2002)	
*Phytophthora infestans*		GIPs	tomato		EGases	Damasceno et al. (2008)	
*Phytophthora infestans*		PC2	EPIs		tomato	P69B	Wang et al. (2021)
Bacteria	*Pseudomonas syringae* pv. *tomato*	Flagellin		galactosyrin	*N. benthamiana*	BGAL1 (FLS2)	Buscaill et al. (2019)
Strategy 7: Disintegrating host-derived hydrolases							
Fungi	*Verticillium dahlia*	Chitin	SSEP1	cotton	Chi28	Han et al. (2019)	
*Fusarium oxysporum f.* sp. *lycopersici*		*Fo*Mep1/Sep1	tomato		Chi1 and Chi13	Jashni et al. (2015)	
*Fusarium verticillioides*		fungalysin	maize		ChitA and ChitB	Naumann et al. (2011)	
*Colletotrichum graminicola*		Cgfl	maize		chitinases	Sanz‐Martín et al. (2016)	
*Colletotrichum cinereus*		fungalysin	N/A		N/A	Lilly et al. (2008)	
*Ustilago maydis*		*Um*Fly1	maize		ZmChiA	Ökmen et al. (2018)	
Level 3: Preventing MAMP perception							
Strategy 8: Degrading MAMPs							
Bacteria	*Pseudomonas aeruginosa* and *Pseudomonas syringae* pv. *tomato*	Flagellin/flg22	AprA	Arabidopsis	(FLS2)	Bardoel et al. (2011)	
Fungi	*Podosphaera xanthii*	Chitin	EWCAs	melon	(CERK1)	Martínez-Cruz et al. (2021)	
Strategy 9: Sequestering released MAMPs							
Fungi	*Cladosporium fulvum*	Chitin	Ecp6	tomato	(CERK1)	De Jonge et al. (2010)	
*Mycosphaerella graminicola*		*Mg*3LysM	wheat		Marshall et al. (2011)		
*Magnaporthe oryzae*		Slp1	rice		Mentlak et al. (2012)		
*Verticillium dahliae* strain VdLs17		Vd2LysM	tomato		Kombrink et al. (2017)		
*Colletotrichum higginsianum*		*Ch*ELP1 and *Ch*ELP2	monocot and dicot crops		Takahara et al. (2016)		
*Rhizoctonia solani*		*Rs*LysM	sugar beet		Dölfors et al. (2019)		
*Trichoderma atroviride*		Tal6	Arabidopsis and tomato		Romero-Contreras et al. (2019)		
*Rhizophagus irregularis*		*Ri*SLM	*Medicago truncatula*		Zeng et al. (2020)		
*Moniliophthora perniciosa*		*Mp*Chi	cacao		Fiorin et al. (2018)		
*Magnaporthe oryzae*		*Mo*Chia1	rice		Yang et al. (2019)		
Bacteria	*Bacillus subtilis*	Flagellin		BSn5	Arabidopsis and voodoo lily	(FLS2)	Deng et al. (2019)

See Supplemental Data Set S1 for a version of this table in excel format.

N/A, nonapplicable.

## Level 1: Three strategies Preventing MAMP production

**Figure 2:**
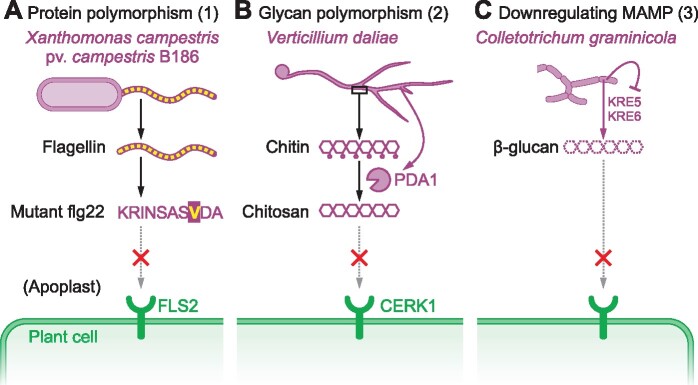
Three mechanisms to avoid MAMP accumulation (Level 1). A, Example of Strategy 1: The bacterial rice pathogen *X. campestris* pv. *campestris* B186 avoids the recognition of its flagellin through an amino acid substitution in the flg22 sequence. B, Example of Strategy 2: the fungal cotton pathogen *V. dahliae* avoids recognition of chitin with secreted polysaccharide deacetylase-1 (PDA1), which converts chitin into chitosan. C, Example of Strategy 3: The fungal maize pathogen *C. graminicola* downregulates the expression of *KRE5* and *KRE6*, which encodes two biosynthesis enzymes required for β-glucan biosynthesis.

We distinguish three different strategies that microbes use to prevent the accumulation of MAMP precursors (Level 1, Figure 1A). The first strategy is to accumulate mutations in protein-based MAMPs to avoid recognition of the MAMP fragment. The second strategy is a similar genetic adaptation to alter glycan-based MAMPs beyond recognition. The third strategy is to downregulate the accumulation of MAMP precursors upon infection. The latter can involve transcription factors, epigenetic regulation, and posttranscriptional control. Specific examples of the three strategies are illustrated in Figure 2.

### Strategy 1: Polymorphisms in protein MAMPs

Polymorphisms in MAMP protein sequences are a frequently used strategy to avoid detection. Sequence polymorphisms have been described for MAMPs from bacterial flagellin, EF-Tu, and RaxX.

In most angiosperms, recognition of bacterial flagellin is mediated by Flagellin Sensing 2 (FLS2), a PRR with extracellular LRRs (Gómez-Gómez and Boller, 2000). FLS2 recognizes peptides from a highly conserved 22-amino acids region in the N-terminal domain of flagellin, called flg22 (Felix et al., 1999; Zipfel et al., 2004). However, some flagellated bacteria carry flg22 sequences that are not recognized by FLS2. flg22 peptides from most ε-, δ-, and α-proteobacteria induce moderate, weak, or no response, respectively, in contrast to flg22 peptides from the majority of γ- and β-proteobacteria, which trigger strong responses (Cheng et al., 2020). For instance, flagellin of the crown gall disease pathogen Agrobacterium (*Agrobacterium tumefaciens*, an α-proteobacterium) possesses a different flg22 sequence that does not trigger FLS2-mediated immune responses in most plant species including Arabidopsis (*Arabidopsis thaliana*; Felix et al., 1999). However, some γ- and β-proteobacteria also evade FLS2 recognition with sequence polymorphism. For example, the bacterial wilt pathogen *Ralstonia solanacearum* (a β-proteobacterium) and some strains of *Xanthomonas campestris* pathovar (pv.) *campestris* (*Xcc*; a γ-proteobacterium) have nonrecognizable versions of the flg22 sequence that evade FLS2-mediated defenses in their respective hosts (Pfund et al., 2004; Sun et al., 2006; Wei et al., 2018). Specifically, an aspartate (D) to valine (V) substitution at amino acid position 14 within the flg22 sequence of flagellin from *Xcc* results in reduced immune responses associated with increased virulence of *Xcc* on Arabidopsis (Figure 2A;Sun et al., 2006). Similarly, *X. oryzae* pv. *oryzae* (*Xoo*) and pv. *oryzicola* (*Xoc*) evade rice (*Oryza sativa*) FLS2-mediated recognition with substitutions in the flg22 sequence (Wang et al., 2015). Consistent with selection for immune evasion, nonpathogenic strains of *X. arboricola* pv. *juglandis* carry the conserved flg22 sequence whereas pathogenic strains carry polymorphisms within the flg22 sequence that evades recognition by FLS2 (Cesbron et al., 2015). Evasion of flagellin recognition by flg22 polymorphisms is also observed with symbiotic bacteria. For instance, the plant beneficial endophytic bacterium *Burkholderia phytofirmans* (a β-proteobacterium) and the plant beneficial bacterium *Sinorhizobium meliloti* (an α-proteobacterium) carry flg22 sequences with weak elicitor activity in grapevine (*Vitis vinifera*) and with no elicitor activity, respectively (Felix et al., 1999; Trdá et al., 2014).

Flagellin is also recognized by FLAGELLIN-SENSING 3 (FLS3), which is present only in some solanaceous plant species including some cultivars of tomato (*Solanum lycopersicum*), potato (*S. tuberosum*), and pepper (*Capsicum annuum*; Hind et al., 2016). FLS3 recognizes flgII-28, a 28-amino acid peptide from the central region of the flagellin protein. Interestingly, polymorphisms within flgII-28 sequences are observed between strains of *Pseudomonas syringae* pv. *tomato* (*Pto*; a γ-proteobacterium). Flagellin of the Col338 strain of *Pto* contains a valine (V) residue at position 13 in the flgII-28 sequence, and this peptide triggers a weaker immune response in tomato than the flgII‐28 peptide from the *Pto*T1 strain, which carries an alanine (A) residue at this position (Cai et al., 2011).

Evasion of recognition caused by flagellin polymorphisms has also been described for animal pathogens. For instance, the human pathogenic bacteria *Campylobacter jejuni*, *Helicobacter pylori*, and *Bartonella bacilliformis* evade flagellin recognition by Toll-Like Receptor 5 (TLR5) with mutations within the key recognition sites of the flagellin N-terminal D1 domain (Andersen-Nissen et al., 2005).

Besides flagellin, evasion with protein polymorphism has also been reported for peptides containing the first 18 amino acids of bacterial Elongation Factor Thermal unstable (EF-Tu), called elf18, which is recognized in Arabidopsis by the EF-Tu Receptor (EFR; Kunze et al., 2004; Zipfel et al., 2006). Polymorphism within the elf18 sequence correlates with different elicitation activity. For instance, elf18 peptides from *Xcc* and *Pto*DC3000 trigger only 0.8%–3.2% of the immune activity as compared to peptides from Agrobacterium, *Ralstonia*, and other *Xanthomonas* and *Pseudomonas* strains (Lacombe et al., 2010).

Another bacterial MAMP with protein polymorphism is the tyrosine-sulfated peptide RaxX, which is perceived by the rice immune receptor XA21 (Pruitt et al., 2015; Luu et al., 2019). XA21 confers resistance to most strains of *X. oryzae* pv. *oryzae* (*Xoo*; Wang et al., 1996). However, *Xoo* isolates carrying nonsynonymous substitutions of tyrosine residue Y41 in RaxX evade XA21-mediated immunity (Pruitt et al., 2015).

In conclusion, protein MAMP polymorphism is an efficient and frequently used strategy to evade host immunity, employed by both pathogenic and symbiotic bacteria. The polymorphisms in MAMPs highlight the exceptional genetic plasticity associated with host adaptation of bacteria. However, amino acid sequence polymorphism is of course restricted by protein function. Flagellin, for instance, cannot accept many substitutions in the flg22 sequence without affecting flagellin function because this region acts as a hinge that facilitates important conformational changes in the flagellin structure when reversing the spin of flagellar rotation (Fliegmann and Felix, 2016; Wang et al., 2017). And obviously, while evasion of immunity by MAMP polymorphism is relevant for protein-based MAMPs, different strategies are needed for nonproteinaceous MAMPs.

### Strategy 2: Polymorphisms in glycan MAMPs

Glycan polymorphism is the second strategy used by microorganisms to evade host immunity. This strategy is illustrated here by the deacetylation of chitin by fungi.

Many fungi evade host immunity by deacetylating chitin into chitosan. Chitin is a structural element of fungal cell walls and chitin fragments are conserved MAMPs that are almost universally recognized in the plant kingdom by Chitin Elicitor Receptor Kinase 1 (CERK1) as a signature of fungal invasion (Pusztahelyi, 2018). Chitosan, however, induces a weaker immune response than chitin, so the deacetylation of chitin is frequently used by plant-associated fungi to avoid recognition (Vander et al., 1998). The soil-borne pathogenic fungus *Verticillium dahliae*, for example, secretes Polysaccharide Deacetylase 1 (*Vd*PDA1) to deacetylate chitin oligomers and prevent chitin-triggered immunity in cotton (*Gossypium hirsutum*) plants (Figure 2B; Gao et al., 2019). *Fusarium oxysporum* f. sp. *vasinfectum* PDA1 is also required for virulence during wilt disease in cotton (Gao et al., 2019). Similarly, the wheat stripe rust fungus *Puccinia striiformis* f. sp. *tritici* suppresses chitin‐induced plant defense by secreting the Polysaccharide Deacetylase Pst13661 (Xu et al., 2020). The wheat (*Triticum aestivum*) stem rust fungus *P. graminis* f. sp. *tritici*, the broad bean (*Vicia faba*) rust fungus *Uromyces fabae*, and the maize (*Zea mays*) anthracnose fungus *Colletotrichum graminicola* also use chitosan instead of chitin in their hyphae (El Gueddari et al., 2002). Likewise, the endophytic fungus *Pestalotiopsis* sp. avoids recognition by secreting chitin deacetylases (*Pes*CDA; Cord-Landwehr et al., 2016). Chitosan is also produced by human pathogens to evade immunity. The human fungal pathogen *Cryptococcus neoformans*, for instance, secretes chitin deacetylase CnCda4 to further deacetylate chitosans that are already partially deacetylated by other chitin deacetylases to evade host immunity (Hembach et al., 2020).

In conclusion, the evolution of glycoforms on the exposed portion of microbes is an efficient strategy to dampen the host immune response. More examples of microbial glycan polymorphisms that evade immunity remain to be discovered. For instance, bacterial pathogens of animals modify the structure of their peptidoglycans (PGNs) through deacetylation and thus evade the antibacterial activity of lysozyme and delay pro-inflammatory immune responses (Boneca et al., 2007; Shimada et al., 2010; Wolf et al., 2011; Grifoll-Romero et al., 2019). However, PGN modification remains to be described for plant pathogens.

### Strategy 3: Downregulating MAMP production

Another microbial strategy to evade host detection is to reduce the amount of MAMPs by downregulating their biosynthesis during infection. This strategy has been described for both bacteria and fungi.

Pathogenic, opportunistic, and commensal bacteria downregulate flagellin biosynthesis during infection. Biosynthesis of flagella in *Pseudomonas* is downregulated by the second messenger cyclic‐di‐GMP (cdG; Hickman and Harwood, 2008). Elevated cdG levels in the plant pathogen *P. syringae*, the plant opportunist *P. aeruginosa* and the plant commensal *P. fluorescens* reduce flagellin levels, and thus contribute to the evasion of FLS2‐mediated immune response in *Nicotiana benthamiana* and Arabidopsis (Pfeilmeier et al., 2016). Flagellin protein levels are also downregulated in *Pto*DC3000 via reduction in flagellin expression by the gene expression regulator AlgU to avoid host immune responses (Bao et al., 2020).

Downregulation of flagellin genes is also observed upon entry of *P. syringae* pv*. syringae* B728a (*Psy*B728a) into the leaf of bean (*Phaseolus vulgaris*) plants (Yu et al., 2013). Comparison of the global transcriptome profiling of *Psy*B728a in epiphytic and apoplastic sites reveals that the mean induction level of genes related to flagellar biosynthesis and motility was >4.5-fold greater on leaf surface than in planta (Yu et al., 2013). Regarding the underlying sensory mechanism to downregulate flagellin expression, *Escherichia coli* downregulates flagellar genes in response to immobilization with anti-flagellar antibodies (Cullender et al., 2013), indicating that flagellin downregulation occurs when bacteria are immobilized.

In fungal pathogens, downregulation of β-glucan biosynthesis reduces exposure to glycan MAMPs. For instance, during the biotrophic phase of infection, the fungal maize pathogen *C. graminicola* downregulates the expression of genes encoding Killer toxin resistant 5 (KRE5) and KRE6, which are key enzymes for the biosynthesis of β‐glucan (Figure 2C;Oliveira‐Garcia and Deising, 2016). However, *KRE5* and *KRE6* expressions are indispensable for the formation of appressoria and necrotrophic hyphae. Consistent with a need for β-glucan, *KRE* genes are also required for full virulence of fungal human pathogens *C. neoformans* and *Candida albicans* (Herrero et al., 2004; Gilbert et al., 2010).

In conclusion, downregulating MAMP precursor levels is a common strategy used by both fungi and bacteria to avoid host detection. Obviously, this strategy is only beneficial for the microbe when production of the MAMP precursor is not required for full virulence. For instance, bacterial proliferation and spread do not rely on flagellin after host entry and the altered fungal cell wall composition may no longer need β-glucans upon host entry.

## Level 2: Preventing MAMP release

**Figure 3:**
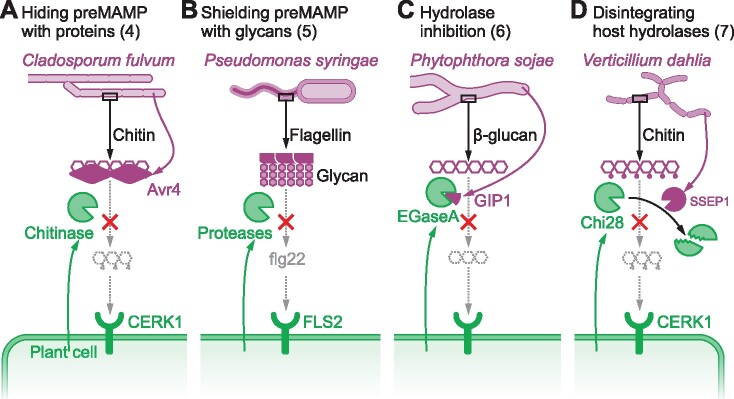
Four mechanisms to avoid hydrolytic MAMP release (Level 2). A, Example of Strategy 4: the fungal tomato pathogen *C. fulvum* prevents the release of chitin fragments by secreting Avirulence protein-4 (Avr4) to hide chitin in the cell wall from hydrolysis by plant-secreted chitinases. B, Example of Strategy 5: the bacterial tobacco (*N. tabacum*) pathogen *Pseudomonas syringae* pv. *tabaci* 6605 prevents the proteolytic release of the flagellin elicitor flg22 by carrying a glycan covering the flagellin polymer. C, Example of Strategy 6: The oomycete soybean pathogen *Phytophthora sojae* prevents the release of β-glucan-based elicitors by secreting glucanase inhibiting protein-1 (GIP1), which inhibits the plant-secreted endoglucanase EGaseA. D, Example of Strategy 7: the fungal cotton pathogen *V. dahlia* prevents the release of immunogenic chitin fragments by Secreting Serine Protease (SSEP1), which inactivates host-secreted chitinase Chi28.

MAMPs discussed in this section are released from microbes by host-secreted hydrolases, such as chitinases and proteases. We distinguish four strategies to block MAMP release from microbes (Figure 1B). MAMP precursors are protected against host hydrolases by microbial proteins and glycans, and host hydrolases are also inhibited and disintegrated. Specific examples of the four strategies are illustrated in Figure 3.

### Strategy 4: Hiding MAMP precursors with proteins

Microbial organisms can evade host recognition by secreting proteins that cover MAMP precursors to prevent hydrolytic release of MAMPs. There are seven examples of this strategy, involving structurally unrelated secreted proteins used by both pathogenic and symbiotic fungi.

The tomato leaf mold fungus (*Cladosporium fulvum* syn. *Passalora fulva*) produces Avr4, a member of Carbohydrate-binding module family 14 (CBM14). Avr4 specifically binds to chitin in the fungal cell wall to protect it against plant chitinases, which release chitin elicitors (Van den Burg et al., 2006; Figure 3A). Homologs of Avr4 are present in other pathogenic fungi, indicating a similar protection of chitin cell walls by other fungi (Stergiopoulos et al., 2010).

Likewise, the hemibiotrophic xylem-invading fungus *V. nonalfalfae* prevents chitin hydrolysis by secreting *Vna*ChtBP, a CBM18 protein that binds chitin and suppresses chitin-triggered host immunity (Volk et al., 2019). *VnaChtBP* is present in 28 *V. nonalfalfae* isolates, suggesting a high evolutionary stability and testifying its importance for the fungal lifestyle.

Similarly, the fungal vascular wilt pathogen *V. dahliae* strain VdLs17 secretes the lineage‐specific LysM effector *Vd*2LysM, a CBM50 protein (Akcapinar et al., 2015), which binds chitin, suppresses chitin‐induced immune responses, and protects fungal hyphae against hydrolysis by plant hydrolytic enzymes (Kombrink et al., 2017). Likewise, the causative fungus of anthracnose diseases, *C. higginsianum*, produces the extracellular LysM proteins 1 and 2 (*Ch*ELP1 and *Ch*ELP2), which bind chitin and prevent chitin-triggered immunity (Takahara et al., 2016).

The wheat Septoria nodorum blotch (SNB) pathogen *Parastagonospora nodorum* secretes SnTox1, a protein that also binds chitin and protects the pathogen from wheat chitinases (Liu et al., 2016). Interestingly, SnTox1 also induces host cell death, supporting the necrotrophic lifestyle of this pathogen (Liu et al., 2012).

Plant beneficial fungi that are parasites of pathogenic fungi also avoid plant immune response by covering MAMPs with proteins. For example, the mycoparasite *Clonostachys rosea* (sin. *Gliocladium roseum*) produces CBM50 members LysM1 and LysM2 to protect hyphae against chitinases to prevent MAMP-induced defenses during wheat root colonization by its host *F. graminearum* (Dubey et al., 2020).

Fungi also prevent MAMP release by hiding β-glucans with proteins. The root endophyte *Serendipita indica* (*Si*, syn. *Piriformospora indica*), avoids recognition of its β‐glucan by secreting a fungal-specific β-glucan-binding lectin, Fungal Glucan-Binding 1 (FGB1). *Si*FGB1 binds β-glucan to reduce β-glucan-triggered immunity in several host plants, including Arabidopsis, barley (*Hordeum vulgare*), and *N. benthamiana* (Wawra et al., 2016).

In conclusion, covering MAMP precursors with proteins to prevent their hydrolysis is a mechanism used by many fungal pathogens and symbionts. Remarkably, several structurally unrelated carbohydrate-binding proteins (Avr4, ChtBP, Vd2LysM, *Ch*ELP1 and *Ch*ELP2, SnTox1, LysM2, and FGB1) have convergently evolved to protect fungal hyphae from hydrolysis by plant chitinases and glucanases, which would otherwise release MAMP from their precursors. In addition to preventing MAMP release, these proteins can also promote virulence by strengthening the cell wall.

### Strategy 5: Shielding MAMP precursors with glycans

Glycosylation of MAMP precursors to shield them from hydrolytic release of MAMPs is the fifth strategy to evade host recognition. For instance, glycosylation of bacterial flagellin and fungal chitin suppress MAMP release.

*O*-glycosylation of flagellin is very common in bacteria, including important plant pathogens from the genera *Xanthomonas*, *Pseudomonas*, *Burkholderia*, *Dickeya*, *Erwinia*, *Pantoea*, and *Pectobacterium* (Taguchi et al., 2010; Ichinose et al., 2013; De Maayer and Cowan, 2016). This flagellin glycosylation is currently thought to prevent the hydrolytic release of the flagellin MAMP (Figure 3B). For instance, glycosylation mutants of *P. syringae* pv. *tabaci* 6605, *P. syringae* pv*. glycinea* race 4 and *X. campestris* pv. *campestris XcA* lacking the flagellin glycosyltransferase (FGT1) are less virulent on tobacco, soybean (*Glycine max*), and cabbage (*Brassica oleracea*), respectively (Takeuchi et al., 2003; Taguchi et al., 2006; Ichinose et al., 2013). Flagellin glycosylation is also important for *Acidovorax avenae*, a Gram-negative bacterial pathogen causing leaf blight in rice. Flagellin isolated from the avirulent N1141 strain induces immune responses, whereas flagellin from the virulent K1 strain does not. These flagellin protein sequences are identical but their glycosylation pattern is different: strain K1 carries a 2,150-Da *O*-glucan while strain N1141 harbors a 1,600-Da *O*-glycan (Hirai et al., 2011). Thus, glycosylation avoids flagellin recognition, presumably by preventing the hydrolytic release of immunogenic flagellin fragments.

Consistent with shielding glycans, plant‐secreted β-galactosidase 1 (BGAL1) acts in immunity by promoting the release of immunogenic peptides from glycosylated flagellin of *Pto*DC3000 and *P. syringae* pv. *tabaci* 6605 (*Pta*6605), which both carry a terminal-modified viosamine (mVio) sugar on flagellin *O*-glycans (Buscaill et al., 2019). BGAL1 is not required to release the flagellin MAMP from the Δ*fgt1* mutant of *Pta*6605, which produces nonglycosylated flagellin. Interestingly, pv. *syringae* B728a (*Psy*B728a) evades host immunity by having *O*-glycans on flagellin that are resistant to hydrolysis by BGAL1 (Buscaill et al., 2019), even though *Psy*B728a has an flg22 sequence recognized by FLS2 (Segonzac et al., 2011). Importantly, mVio biosynthesis genes are absent from *Psy*B728a, which instead carries a putative (1,2)-linked terminal GlcNac (*N*-acetylglucosamine) on its *O*-glycan (Yamamoto et al., 2011; Chiku et al., 2013). This suggests that different glycoforms on flagellin are required for the colonization of different hosts and that hosts may use different glycosidases to release flagellin MAMPs.

Bacteria also use polymorphic lipopolysaccharides (LPSs) to evade immunity. LPSs are the major component of the outer membrane of Gram-negative bacteria and consist of lipid A, a di-glucosamine carrying four to seven fatty acids, and an oligosaccharide core region that carries an *O*-polysaccharide (OPS) comprising a variable number of oligosaccharide repeats. OPS composition is highly diverse among bacterial species and strains. Glycans covering bacterial LPS may alter host responses. For instance, the plant pathogenic bacterium *Xylella fastidiosa* possesses a long chain *O*-antigen that delays recognition by the host plant (Rapicavoli et al., 2018). Mutant *X. fastidiosa* lacking these *O*-antigens induces faster immune responses (Rapicavoli et al., 2018). In addition, genes that encode glycosyltransferase domains and cause strong virulence phenotypes when disrupted in *Psy*B728a are suspected to be involved in the biosynthesis of *O*-antigens that decorates LPS (Helmann et al., 2019).

Glycosylation of fungal cell walls is also used to prevent MAMP release. For instance, the rice blast fungus *Magnaporthe oryzae*, the rice brown spot fungus *Cochlioborus miyabeanus*, and the rice sheath blight fungus *Rhizoctonia solani* accumulate α-1,3-glucan on the surface of infectious hyphae (Fujikawa et al., 2009, 2012). Fungal mutants with reduced α-1,3-glucan levels have reduced virulence, indicating that α-1,3-glucan may mask chitin and β-glucans in the fungal cell wall to shield it against hydrolytic MAMP release (Fujikawa et al., 2012). The large phylogenetic distance between these ascomycete and basidiomycete rice pathogens indicates that this strategy is a widespread mechanism that may be used by fungal pathogens to evade host innate immunity.

Glycan shielding of MAMP precursors has also been described for human pathogens, including viruses, fungi, and bacteria, as a strategy to evade recognition by the host immune system (Walls et al., 2016; Hernández-Chávez et al., 2017; Poole et al., 2018; PradHan et al., 2019). For instance, flagellin glycosylation reduces recognition of human bacterial pathogens, providing an evasive strategy for *P. aeruginosa* (Arora et al., 2005) and *B. cenocepacia* (Hanuszkiewicz et al., 2014). In conclusion, glycan shielding of MAMP precursors is a common strategy used by many pathogens to enhance infection. These findings predict that many more examples of glycan shielding will be discovered for plant pathogens and symbionts.

### Strategy 6: Blocking MAMP release by inhibiting the activity of host hydrolases

The inhibition of MAMP-releasing hydrolases is another strategy used by plant pathogens. Examples of this strategy have been identified in oomycetes and bacterial pathogens.

*Phytophthora* species secrete glucanase inhibitor proteins (GIPs) during invasion of their hosts, which themselves inhibit MAMP release through extracellular Endo-β-1,3-Glucanases (EGases). For example, *P. sojae* secretes GIP1 to inhibit soybean EGaseA, thereby preventing EGaseA-mediated release of elicitor-active glucan oligosaccharides (Figure 3C; Rose et al., 2002). Analysis of tomato leaves inoculated with *P. infestans* showed that *P. infestans* GIPs and tomato EGases form stable complexes in the apoplast (Damasceno et al., 2008), indicating that GIPs-mediated inhibition of EGases to prevent MAMP release is a common strategy used by *Phytophthora* in different hosts.

*Phytophthora* species also produce Kazal-like Extracellular Protease Inhibitors (EPIs) during infection. *P. infestans* EPI1 and EPI10 inhibit the secreted subtilisin-like protease P69B of tomato (Tian et al., 2004; Tian et al., 2005). P69B releases a fragment from the apoplastic effector PC2 of *P. infestans* that then triggers immune responses and a hypersensitive response (HR) in solanaceous plants (Wang et al., 2021). Thus, EPI1 might suppress PC2-elicited host immunity by inhibiting the protease that releases the elicitor.

Bacterial pathogens also produce hydrolase inhibitors to prevent MAMP release. For instance, *Pto*DC3000 produces the small molecule BGAL1 inhibitor galactosyrin (Buscaill et al., 2019). BGAL1 promotes the release of MAMPs from glycosylated flagellin carrying mVio on their *O*-glycan (see Strategy 5). BGAL1 is suppressed by galactosyrin during infection to prevent the release of immunogenic peptide from flagellin (Buscaill et al., 2019).

GIP1, EPI1, and galactosyrin are just the first examples of pathogen-derived inhibitors targeting MAMP-releasing host hydrolases. Further studies on widespread pathogen-derived inhibitors will probably uncover many more examples. However, in addition to preventing MAMP release, these inhibitors also protect the physical integrity of flagellin and the microbial cell wall by preventing their degradation.

### Strategy 7: Disintegrating host-derived hydrolases

Destruction of MAMP-releasing host hydrolases is the seventh strategy used by invading microorganisms to evade immunity. We currently know three unrelated classes of proteases from fungal pathogens that disintegrate host chitinases to prevent MAMP release.

The root-infecting fungal pathogen *V. dahlia* produces Secreted Serine Protease 1 (SSEP1, family S8) during the invasion of cotton root cells to hydrolyze cotton Chitinase 28 (Chi28; Figure 3D; Han et al., 2019). Likewise, the vascular wilt pathogen of tomato, *F. oxysporum f.* sp. *lycopersici*, secretes family-M36 metalloprotease fungalysin *Fo*Mep1 and family-S8 subtilisin-like protease *Fo*Sep1 to remove the extracellular chitin-binding domain (CBD) from tomato chitinases *Sl*Chi1 and *Sl*Chi13 (Jashni et al., 2015). Removal of the CBD significantly reduces chitinase and antifungal activity, thereby playing a pivotal role in virulence of *F. oxysporum*. Similarly, the fungal pathogens *F. verticillioides*, *C. graminicola*, *Coprinopsis cinerea*, and *Ustilago maydis* also secrete family-M36 metalloprotease fungalysins to cleave plant chitinases (Lilly et al., 2008; Naumann et al., 2011; Sanz‐Martín et al., 2016; Ökmen et al., 2018). Interestingly, animal fungal pathogens also produce fungalysin during infection, presumably with the same objective (Li and Zhang, 2014).

In conclusion, SSEP1, *Fo*Sep1, *Fo*Mep1, and other fungalysins represent different protease families that cleave plant chitinases to prevent MAMP release. The phylogenetic distance between these fungi and proteases indicates that the inactivation of chitinases evolved convergently. Besides preventing MAMP release, these proteases also protect the physical integrity of the cell wall by preserving chitin.

## Level 3: Preventing MAMP perception

**Figure 4:**
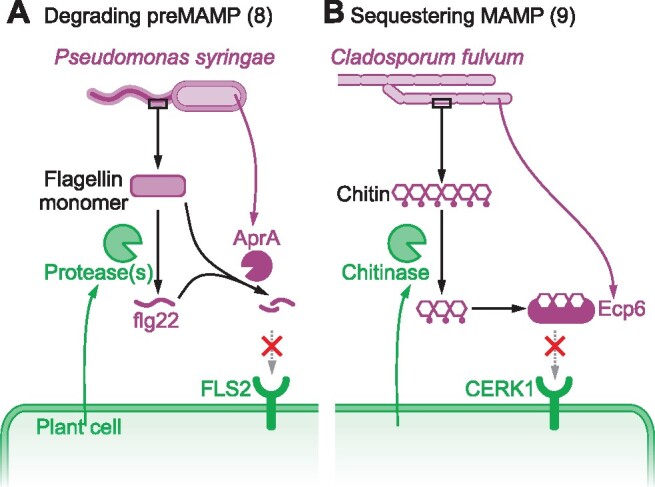
Two mechanisms to prevent MAMP perception (Level 3). A, Example of Strategy 8: the bacterial plant pathogen *Pseudomonas syringae* prevents the accumulation of immunogenic flagellin fragments by secreting the metalloprotease AprA, which can cleave both the flagellin monomer and the flg22 elicitor peptide. B, Example of Strategy 9: The fungal tomato pathogen *C. fulvum* prevents the accumulation of immunogenic chitin fragments by secreting Extracellular cysteine-rich protein-6 (Ecp6), which sequesters chitin fragments.

Once MAMPs are released, we know two effective strategies that prevent the perception of MAMPs by PRRs (Figure 1C). One strategy degrades MAMPs before they reach their receptors, the other strategy sequesters MAMPs before they are perceived. Specific examples of the two strategies are illustrated in Figure 4.

### Strategy 8: Degrading MAMPs

The eighth strategy used by pathogens is based on the targeted degradation of released MAMPs by pathogen-derived proteases. Examples are the bacterial effectors AprA and LasB and fungal effectors with chitinase activity (EWCAs), explained below.

*Pseudomonas* species secrete alkaline protease ArpA, a 50-kD zinc metalloprotease (MEROPS family M10 of clan MA), which prevents flagellin-triggered immune responses by degrading flagellin monomers and flg22 (Figure 4A; Bardoel et al., 2011). Flagellin polymers resist degradation by AprA and this preserves the integrity of the flagellum (Bardoel et al., 2011). In Arabidopsis, AprA prevents flg22- and flagellin-induced immune responses and delays stomatal closure. AprA of *Pto*DC3000 is required for its full virulence on both Arabidopsis and tomato. Interestingly, AprA is widespread among human- and plant-pathogenic bacteria, including the bacterial plant pathogen *P. syringae* and human pathogen *P. aeruginosa*, suggesting a conserved infection mechanism among bacteria.

In addition to AprA, *P. aeruginosa* secretes a second zinc metalloprotease, the 33-kD pseudolysin LasB (MEROPS family M4 of clan MA), which also degrades flagellin and acts in concert with AprA to prevent flagellin-mediated immune recognition (Casilag et al., 2016). The production of two different proteases targeting flagellin monomers likely provides *P. aeruginosa* with robust immune suppression mechanisms.

Fungal pathogens also degrade MAMPs during infection. The cucurbit powdery mildew fungus *Podosphaera xanthii* releases effectors with chitinase activity (EWCAs) when penetrating melon (*Cucumis melo*) plant cells to degrade immunogenic chitin oligomers and thereby prevents the activation of chitin-triggered immunity (Martínez-Cruz et al., 2021). Remarkably, *EWCA* homologs are also widely distributed in plant fungal pathogens but also in fungi that are pathogens of insects, nematodes, and animals, suggesting a conserved infection mechanism among fungi (Martínez-Cruz et al., 2021).

In conclusion, the degradation of MAMPs is an efficient mechanism to avoid recognition but only a few of these proteases have been identified. Additional pathogen-produced proteases that promote virulence (Chandrasekaran et al., 2016; Figaj et al., 2019) may also act by degrading MAMPs. Likewise, pathogen-secreted glycosidases may degrade glycan-based MAMPs. For instance, the human pathogen *Histoplasma capsulatum* secretes endo-β-1,3-glucanase Eng1 to evade host detection (Garfoot et al., 2016) and many plant pathogens having glycan-based MAMPs secrete glycosidases (Ospina-Giraldo et al., 2010; Murphy et al., 2011; Vermassen et al., 2019; McGowan et al., 2020). Notably, MAMP degradation must require fine regulation of these hydrolases to avoid unspecific or premature degradation of MAMP precursors or inadvertent MAMP release.

### Strategy 9: Sequestering released MAMPs

Elicitor sequestration is the ninth strategy used by plant pathogens to evade recognition. In this strategy, pathogen-secreted proteins bind released elicitors to prevent them from binding host receptors. Many fungi secrete proteins to sequester chitin elicitors.

During infection, *C. fulvum* secretes Extracellular protein 6 (Ecp6), a LysM-containing protein of the CBM50 family that binds chitin fragments. Ecp6 binds these elicitors with greater affinity than the chitin receptor, so *C. fulvum* evades chitin recognition by using Ecp6 to sequester chitin fragments, (Figure 4B; De Jonge et al., 2010). The widespread presence of Ecp6 orthologs suggests that this is a common strategy of many pathogenic fungi to avoid host recognition (De Jonge and Thomma, 2009). Indeed, the fungal wheat pathogen *Mycosphaerella graminicola* produces *Mg*3LysM, an Ecp6 homolog, that plays a major role in pathogen virulence on wheat plants by preventing the elicitation of chitin-induced immunity (Marshall et al., 2011). Similarly, the rice blast fungus *M. oryzae* produces Secreted LysM protein-1 (Slp1), which binds chitin fragments and prevents chitin-triggered immunity (Mentlak et al., 2012). Interestingly, *Vd*2LysM from *V. dahlia* and *Ch*ELP1 and *Ch*ELP2 from *C. higginsianum*, also sequester chitin oligomers and additionally protect chitin polymers against chitinases (Strategy 4; Takahara et al., 2016; Kombrink et al., 2017).

Symbiotic fungi also use LysM proteins to establish compatible interactions. The endophytic fungus *Trichoderma atroviride* and the arbuscular mycorrhiza fungus *Rhizophagus irregularis*, for instance, produce the LysM proteins Tal6 and *Ri*SLM, respectively, to evade extracellular recognition (Romero-Contreras et al., 2019; Zeng et al., 2020). Surprisingly, even necrotrophic fungi use LysM proteins to evade immunity. For instance, the necrotrophic fungus *R. solani*, which kills seedlings and causes root rot in a broad range of plant species, secretes *Rs*LysM, which associates with chitin oligomers to prevent early chitin perception during sugar beet (*Beta vulgaris*) colonization (Dölfors et al., 2019).

LysM proteins are also used by animal fungal pathogens and contribute to fungal virulence during host invasion. The insecticidal fungus *Beauveria bassiana* secretes the two LysM effectors Blys2 and Blys5 that bind chitin and prevent the activation of immunity in insects (Cen et al., 2017).

Sequestration of chitin fragments is also achieved through different evolutionary paths. The cacao (*Theobroma cacao*) witches broom disease *Moniliophthora perniciosa* produces an enzymatically inactive chitinase (*Mp*Chi) that prevents chitin-triggered immunity by sequestering chitin fragments (Fiorin et al., 2018). Remarkably, its sister species *M. roreri* encodes a second, nonorthologous catalytically inactive chitinase (*Mr*Chi). *MpChi* and *MrChi* are both highly expressed during the biotrophic phase of infection. Despite lacking chitinolytic activity, both proteins sequester immunogenic chitin fragments. Similarly, the fungal rice pathogen *M. oryzae* secretes Chitinase 1 (*Mo*Chia1) that binds chitin and can prevent immune responses (Yang et al., 2019).

Bacteria also use the sequestration strategy by targeting MAMP precursors. For instance, the endophyte bacterium *Bacillus subtilis* BSn5 enhances its colonization of Arabidopsis and voodoo lily (*Amorphophallus konjac*) through minimizing the stimulation of flg22-induced defense by producing the antimicrobial peptide (lantibiotic) subtilomycin, which binds to its own flagellin (Deng et al., 2019). The presence of subtilomycin biosynthesis genes in genomes of other bacteria suggests that flagellin sequestration is a common strategy of endophytic bacteria to adapt to endosphere niches (Deng et al., 2019).

MAMP sequestration is common to fungal and bacterial microbes, but more details remain to be discovered in other invading microorganisms. The success of this strategy depends on the competition between the microbial-derived sequestering protein and the host PRR.

## Concluding remarks and perspectives

Successful plant-associated microbes evade extracellular detection by the host immune system. While certain immune evasion mechanisms are used by microbes from diverse kingdoms, other mechanisms have so far only been described for certain microbes. However, it seems unlikely that these strategies are pathogen-specific, prompting us to expect that this is likely to change with further development of the field.

For most of the nine strategies described above, suppression MAMP perception also results in increased stability of the MAMP precursor. It has therefore not always been robustly demonstrated that the observed enhanced virulence associated with the strategy is caused by evading MAMP recognition or by increased stability of the MAMP precursor. A good way to investigate this experimentally would be to test for enhanced virulence in the absence of the PRR, as this would indicate an important role in the stabilization of the MAMP precursor.

We can think of at least three additional extracellular strategies that would prevent MAMP recognition (Figure 1D). First, MAMP recognition can be blocked by receptor shedding. This has been described for animal pathogens, but not for plant pathogens. For example, the fungal respiratory pathogen *Coccidioides posadasii* secretes the Metalloproteinase Mep1 during endospore differentiation. Mep1 digests the Spherule Outer Wall glycoprotein (SOWgp), resulting in the prevention of host recognition mediated by this receptor (Hung et al., 2005). Similarly, the opportunistic pathogen of human lungs *P. aeruginosa* secretes the metalloproteinase LasB, which cleaves the human urokinase-type Plasminogen Activator Receptor (uPAR) through domain-specific endoproteolysis (Leduc et al., 2007). There have been no reports of PRR inactivation by shedding in plant-microbe interactions yet. By contrast, ectodomain shedding of the legume Symbiosis Receptor Kinase (SYMRK) causes the formation of a signaling complex with Nod Factor Receptor 5 (NFR5, Antolín-Llovera et al., 2014).

A second possible strategy is the use of antagonists. MAMP antagonists would bind PRRs and prevent the binding of MAMPs to their respective receptors and therefore inhibit PRR function. For instance, C-terminal truncations of the flagellin flg22 elicitor can block flg22 perception by FLS2 in tomato (Meindl et al., 2000), but the existence and use of MAMP antagonists during infection remains to be reported.

A third possible strategy is to alter the apoplastic conditions such that MAMP release and/or perception is inhibited. Although this mechanism has not yet been demonstrated, the regulation of hydrolases by pH and redox status would offer opportunities for pathogens to interfere in MAMP release. Interestingly, several plant pathogens secrete homologs of plant regulatory peptides to suppress host immunity. For instance, the fungal wilt pathogen *F. oxysporum* secretes a functional homolog of rapid alkalinization factors (RALFs), peptide hormones that trigger an increase of apoplastic pH and enhances fungal colonization (Masachis et al., 2016; Thynne et al., 2017).

In conclusion, the extracellular detection of MAMPs by plants is an active and exciting research field. The presence of many MAMPs, hydrolytic enzymes, and hydrolase inhibitors implicate a large and mostly unexplored area of research, still holding most of its secrets. Increased understanding of this extracellular battlefield of both animal and plant pathogens will ultimately translate into new strategies for the prevention of infectious diseases.

## Supplemental data

The following materials are available in the online version of this article.

**Supplemental Data Set S1.** Overview of strategies employed by microbes to evade MAMP recognition in plants.

## Supplementary Material

koab109_Supplementary_Data

## Notes

The authors wrote the article together. The author responsible for distribution of materials integral to the findings presented in this article in accordance with the policy described in the Instructions for Authors (https://academic.oup.com/plcell) is: Renier A. L. van der Hoorn (renier.vanderhoorn@plants.ox.ac.uk).
